# Reply to Wade and Laub, ‘Concerns about “Stress-Induced MazF-Mediated Proteins in Escherichia coli”’

**DOI:** 10.1128/mBio.01063-19

**Published:** 2019-06-04

**Authors:** Akanksha Nigam, Tamar Ziv, Adi Oron-Gottesman, Hanna Engelberg-Kulka

**Affiliations:** a Department of Microbiology and Molecular Genetics, IMRIC, The Hebrew University Hadassah Medical School, Jerusalem, Israel; b Smoler Proteomics Center, Faculty of Biology, Technion, Haifa, Israel; Harvard University

**Keywords:** bacterial stress response, proteomics, toxin-antitoxin

## REPLY

We thank Dr. Wade and Dr. Laub for taking the time to offer comments regarding our paper “Stress-Induced MazF-Mediated Proteins in Escherichia coli,” but we disagree with the points raised by them, and we answer all the questions as follows.

In response to the first concern raised by Dr. Wade and Dr. Laub, namely, that there is no significant enrichment of ACA sequences upstream of MazF-induced genes, we did not claim that “there is a significant enrichment of ACA sequences upstream of MazF-induced genes.” Rather we claim that MazF-induced genes carry an ACA sequence in the region 100 nucleotides upstream of the AUG initiator of their mRNAs. We did not use the phrase “enrichment of ACA sequences.” Furthermore, as noted our criticized paper (1), our results corroborated previous findings (2) showing that in E. coli, the stress-induced MazF-mediated stress translation machinery (STM) is composed of deficient ribosomes (3) and mRNAs carrying the MazF cleavage site ACA up to 100 nucleotides upstream of the AUG initiation codon (2). The word “remarkably” was understood by Dr. Wade and Dr. Laub in the wrong way. As written in our criticized paper (1), “Remarkably, with the exception of six of them, in all the corresponding mRNAs specifying these proteins, the MazF cleavage site ACA was found to be located up to 100 nucleotides upstream of the AUG initiator” (Results, first paragraph, line 23 of the PDF version). We use “remarkably” for our results to show that most of the stress-induced MazF-mediated proteins (36 out of 42) which were detected in our study (1) carry the MazF cleavage site, ACA, up to 100 nucleotides upstream of the AUG initiator of their mRNAs and not that each of their mRNAs carries a significant enrichment of the ACA sequence.

In response to the second concern raised by Dr. Wade and Dr. Laub, namely, that there is insufficient evidence to support a role for MazF in ACA-mediated regulation, we state the following. (i) Dr. Joseph T. Wade and Dr. Michael T. Laub claim that “this experiment lacks a critical control to show that the effects of NA treatment are dependent upon MazF.” They also claim that “Nigam et al. did not determine whether the effects of ACA location on NA-dependent changes in GFP expression were lost in a strain lacking *mazF*.” However, as mentioned in the criticized paper (1), the GFP construct used in our study was well established in a previous paper of ours (4), where we showed that the described GFP reporter is expressed only under stress (NA) in the wild-type (WT) strain and not in its *ΔmazEF* derivative. (ii) In that previous paper of ours (4), we had also already shown that the GFP construct is suitable for this research. A schematic presentation of the GFP–stress-induced translation machinery (STM) reporter is illustrated in Fig. 1G of that paper (4). It was shown to carry an ACA codon located immediately upstream of AUG, appearing as ACAUG, which is called a leaderless mRNA. Upstream of this leaderless sequence, we inserted a stem and loop structure in order to avoid Shine-Dalgarno (SD) base pairing and thereby avoid translation by the canonical translation machinery. Although the mRNA of this GFP reporter carries ACAs, they are all out of frame and therefore not cleaved by MazF, as reported by Oron-Gottesman et al. (4). It is actually the main issue of our previous paper (4) that MazF (induced under stress) cleaves ACAs in mRNA that are located in frame 0 and not in frames +1 and +2. Thus, in our previous paper (4), we had already shown that the GFP construct is suitable for the research of the criticized paper (1). In our previous study (4), we also used another reporter, this time an endogenous reporter, *groEL*, which is heat shock and *mazF* dependent and which has all of its ACAs located out of frame. Therefore, GFP is a suitable reporter for studying the stress translation machinery described in the criticized paper (1).

We are now in the process of showing that heat shock protein σ^32^ is itself a product of the STM. It has an ACA located in the 100-nucleotide region upstream of the AUG initiator, its expression depends on *mazF*, and it is a well-known heat shock protein and therefore should be induced by *mazF*. Sigma 32 was not obtained in our proteomic study (1) because it is degraded by Lon protease, and our experiments were not done by using a Lon-deficient derivative.

The two papers in references 5 and 6 are not cited by Nigam et al. because, according to our expertise in the field, wrong methods were used by their authors. In fact, our criticized paper (1) provides proof from proteomic studies for the existence of the STM system.
